# Therapeutic Effects of Phytochemicals and Medicinal Herbs on Chemotherapy-Induced Peripheral Neuropathy

**DOI:** 10.3390/molecules21091252

**Published:** 2016-09-20

**Authors:** Gihyun Lee, Sun Kwang Kim

**Affiliations:** 1 Department of Physiology, College of Korean Medicine, Kyung Hee University, 26 Kyunghee-daero, Dongdaemoon-gu, Seoul 02447, Korea; glee@khu.ac.kr; 2 Department of Research and Development, National Development Institute of Korean Medicine, 94 Hwarang-ro, Gyeongsan-si, Gyeongsangbuk-do 38540, Korea

**Keywords:** phytochemical, medicinal herb, chemotherapy-induced peripheral neuropathy

## Abstract

Chemotherapy-induced peripheral neuropathy (CIPN) is a frequent adverse effect of neurotoxic anticancer medicines. It leads to autonomic and somatic system dysfunction and decreases the patient’s quality of life. This side effect eventually causes chemotherapy non-compliance. Patients are prompted to seek alternative treatment options since there is no conventional remedy for CIPN. A range of medicinal herbs have multifarious effects, and they have shown some evidence of efficacy in various neurological and immunological diseases. While CIPN has multiple mechanisms of neurotoxicity, these phytomedicines might offer neuronal protection or regeneration with the multiple targets in CIPN. Thus far, researchers have investigated the therapeutic benefits of several herbs, herbal formulas, and phytochemicals in preventing the onset and progress of CIPN in animals and humans. Here, we summarize current knowledge regarding the role of phytochemicals, herb extracts, and herbal formulas in alleviating CIPN.

## 1. Introduction

Several antineoplastic medicines are reported to cause neurotoxicity and can develop chemotherapy-induced peripheral neuropathy (CIPN) [1]. These drugs have effects on sensory nerves and cause substantial pain, dysfunction, and finally chemotherapy non-compliance [2,3]. This adverse effect damages peripheral nerves and can lead to sensory deficits, gait impairment [4], or severe neuropathic pain [5], and can severely degrade the patient’s quality of life [6]. The most common symptoms reported by patients include sensory symptoms such as numbness, burning, tingling, throbbing, and burning feelings. Moreover, patients may experience motor symptoms, such as dropping items, splaying fingers, and inability to complete normal daily activities [7,8]. 

CIPN is difficult to prevent and control without dose-reduction or cessation of anticancer drugs [9]. The overall incidence of this adverse effect is remarkably high [10], although the population affected depends on chemotherapy drugs, dose, and exposure time [11,12]. Usually the symptoms of CIPN are reversible; however, sometimes symptoms are irreversible [13] and worsen after withdrawal of drugs, including vincristine, cisplatin, oxaliplatin, or paclitaxel [14,15,16].

Thus far, various pharmacological tactics have been attempted to attenuate CIPN symptoms. These medications include acetyl-l-carnitine, amifostine, glutathione, glutamine, vitamin E, PARP inhibitors, leukemia inhibitory factor, *N*-acetylcysteine, Ca/Mg, and venlafaxine [17,18,19]. The therapeutic potentials of these drugs are limited by unexpected adverse effects and contradictory results, although these drugs have shown benefits in preventing CIPN [20,21]. Still, no approach has sufficient evidence for recommending use in CIPN treatment. Hence, alternative methods of preventing or treating CIPN are necessary.

Medicinal herbs have been used as therapeutics for centuries throughout the world. Phytochemicals derived from these medicinal plants are used to treat various neurological and immunological disorders. On the basis of recent literature, several phytochemicals, herbs, and herbal formulas exhibiting promising effects on CIPN have been identified. Here, we summarize the therapeutic effects of phytochemicals (see also Table 1), medicinal herbs (Table 2), and herbal formulas (Table 3) against CIPN induced by vincristine, cisplatin, oxaliplatin, and paclitaxel. 

## 2. Phytochemicals and Medicinal Herbs against Vincristine-Induced Peripheral Neuropathy

Vincristine is one of the most extensively used chemotherapeutic agents to treat diverse types of cancer, including Hodgkin‘s disease, small cell lung cancer, acute myeloid leukemia, acute lymphocytic leukemia, and neuroblastoma. Vincristine inhibits chromosome separation during the metaphase resulting in cell apoptosis [49]. Patients can experience some side effects from vincristine treatment, such as headaches, hair loss, walking difficulty, constipation, and a change in sensation. In serious cases, neuropathic pain, classically resulting in autonomic and peripheral sensory-motor neuropathy limits the dose of vincristine. Vincristine-induced peripheral neuropathy can worsen after therapy has ended [50].

### 2.1. Acorus calamus

*Acorus calamus* is a medicinal herb used to alleviate pain or severe inflammation in Ayurveda. The root of the plant is widely used to treat a number of illnesses such as abdominal tumors, chronic diarrhea, dysentery, epilepsy, fever, mental ailments, kidney and liver issues, and rheumatism [51]. Hydro-alcoholic extracts of *A. calamus* rhizoma (100–200 mg/kg, p.o. for 14 consecutive days) protect against painful neuropathy induced by vincristine in rats. The extracts inhibit vincristine-induced biochemical (increase in superoxide anion generation level and total calcium level, and myeloperoxidase activity in the sciatic nerve) and behavioral (thermal- and mechano-hyperalgesia) changes to an extent comparable to Lyrica (pregabalin) [28]. The ethanolic extract of *A. calamus* (up to 600 mg/kg) did not cause lethality and any changes in the general behavior in rats in both acute and chronic toxicity tests [52].

### 2.2. Auraptenol

Auraptenol (8-(2-hydroxy-3-methylbut-3-enyl)-7-methoxychromen-2-one) is a phytochemical isolated from *Angelicae dahuricae radix*. The root of the plant is used to treat harmful exterior stimuli on the skin, such as dryness, dampness, heat, and cold in Oriental medicine [53]. It has been shown that its antinociceptive effects are linked to the facilitated release of endogenous opioids [54] and that a single oral administration of *A.* *dahuricae* (3.25 g or 6.5 g) decreased cold-induced tonic pain in a dose-dependent manner in clinical trials [55]. It reverted mechanical hyperalgesia induced by vincristine through 5-HT_1A_ receptors in a dose-dependent manner (within the 0.05–0.8 mg/kg range). The highest dose of auraptenol (0.8 mg/kg, i.p.) totally suppressed the mechanical hyperalgesia without affecting the general locomotor activity [22].

### 2.3. Butea monosperma

*Butea monosperma* is a medium-sized deciduous tree that has been used as an aphrodisiac, astringent, tonic, and diuretic in Ayurveda [56]. The plant contains many phytocomponents, including saponins, glycosides, mucilage, gums, and fatty acids [57]. Oral intake of ethanolic extract of *B. monosperma* (200–400 mg/kg for 14 days) suppressed histological, biochemical, and behavioral changes induced by vincristine in rats. Authors have suggested that the therapeutic benefits might originate from its calcium channel inactivating, anti-oxidative, anti-inflammatory, and neuroprotective effects [29].

### 2.4. Cannabinoids

Historically, *Cannabis sativa* has used to treat neuropathic pain. Cannabinoids repress neurotransmitter release in the brain by binding on cannabinoid receptors in cells [58] and have anti-inflammatory effects [59]. Rahn et al. investigated the effect of synthetic Δ^9^-tetrahydrocannabinol analog on vincristine-induced mechanical allodynia in rats. The experiment demonstrated that cannabinoids can inhibit vincristine-induced mechanical allodynia through the cannabinoid receptor 1/2 pathway and the effect is mediated at the level of the spinal cord in part, although these synthetic cannabinoids may have some different pharmacological effects from phytocannabinoids [60]. Cannabinoids have shown anti-cancer activities in animal models of cancer, and they are currently being tested as anti-tumor agents in phase I/II clinical trials [61].

### 2.5. Ginkgo biloba L.

*Ginkgo* leaf extract has been used for pharmaceutical purposes since 1965 and is one of the bestselling herbal medicines in the world [62]. Park et al. showed that *Ginkgo biloba* extract (50–150 mg/kg, p.o.) decreased the paw withdrawal frequency to cold stimuli and increased the withdrawal threshold to mechanical stimuli in peripheral neuropathy-induced rats [30]. They suggested that the anti-hyperalgesic effect of *G. biloba* extract may be related to suppression of axonal degradation, improved axonal transport, and inhibition of TNF-α and NO production. The few recent studies on the anticancer activity of the extract in in vitro models showed the cell proliferation inhibition, tumor suppression, and DNA damage-repairing effects of the extract [63,64,65]. Biggs et al. analyzed the risk of cancer hospitalization between participants assigned to *Ginkgo* extract treatment and those assigned to placebo and reported that the data do not support the regular use of *G. biloba* for reducing the risk of cancer [66].

### 2.6. Ocimum sanctum L.

In traditional medicine, *Ocimum sanctum* L. has been recommended for the treatment of skin diseases, bronchitis, diarrhea, malaria, and arthritis. Recent research has also demonstrated its cardioprotective, anti-microbial, anti-fungal, anti-fertility, anti-diabetic, anti-cancer, and analgesic properties [67]. Its leaf oil contains eugenol, eugenic acid, ursolic acid, carvacrol, linalool, limatrol, caryophyllene, and methyl carvacrol [68]. Kaur et al. demonstrated that oral administration of *O. sanctum* or its saponin-rich fraction (100 and 200 mg/kg, for 14 days) reduced neurotoxicity induced by vincristine in rats with a decline in calcium levels and oxidative stress, thus helping to prevent CIPN symptoms. They estimated that the saponin-rich fraction may mediate the therapeutic effects of *O. sanctum* in neuropathic pain [33]. Seed oil supplementation of *O. sanctum* L. (100 μL/kg) reduced 20-methaylcholathrene-induced tumor incidence and tumor volume and enhanced the survival rate in mice [69].

### 2.7. Xylopic Acid

Traditionally, the fruit of *Xylopia aethiopica* has been used to manage pain disorders, including neuralgia and headache [70]. Ethanolic extract of *X. aethiopica* (30–300 mg/kg, p.o.) and its major diterpene xylopic acid (15-(acetyloxy)kaur-16-en-18-oic acid; 10–100 mg/kg, p.o.) exhibit anti-allodynic and anti-hyperalgesic properties in vincristine-induced neuropathic pain. Diterpene xylopic acid from *X. aethiopica* exhibited greater potency than the ethanolic extract of *X. aethiopica* itself, while pregabalin (10–100 mg/kg) showed a comparable effect to xylopic acid [27]. Treatment with *X. aethiopica* extract led to a dose-dependent growth inhibition in many cell lines, including HCT116 colon cancer cells, U937, and KG1a leukemia cells, and the C-33A cervical cancer cell line [71,72], but xylopic acid, unlike kaurenoic acid, has no cytotoxic effects on human cancer cells [73].

## 3. Phytochemicals and Medicinal Herbs against Cisplatin-Induced Peripheral Neuropathy

Cisplatin is the first member of platinum-based antineoplastic medicine. This platinum complex causes the crosslinking of two DNA strands in cells, which prevents cell division and finally leads to programmed cell death. In addition to nephrotoxicity, neurotoxicity and ototoxicity are dose-limiting adverse effects of cisplatin treatment [74,75].

### 3.1. Curcumin

Curcumin ((1*E*,6*E*)-1,7-bis(4-hydroxy-3-methoxyphenyl)-1,6-heptadiene-3,5-dione) is a yellow pigment component of *Curcuma longa*. This phytochemical is well known for its powerful anti-inflammatory and antioxidant properties. It has demonstrated benefits in neuronal diseases such as alcoholic neuropathy and diabetic neuropathy [76,77,78]. In the cisplatin-treated model, curcumin (10 mg/kg, oral) reversed the neurotensin changes in the plasma, reduced cisplatin absorption in the sciatic nerve, and notably ameliorated sciatic nerve histology [24]. Curcumin regulates the growth of cancer cells by the modulation of multiple cell signaling pathways, including protein kinase, mitochondrial, death receptor, caspase activation, cell survival, tumor suppressor, and cell proliferation pathways [79]. Extensive in vivo data support curcumin’s beneficial effects against cancer [80,81,82]; however, there are also conflicting reports that curcumin can promote cancer in mice [83,84].

### 3.2. Ginkgo biloba L.

Ozturk et al. showed that oral administration of *G. biloba* alcoholic extract is beneficial in preventing peripheral neuropathy induced by cisplatin in mice. In their experiments, *G. biloba* extract reduced cisplatin-induced immigrated cell numbers, sensory nerve conduction velocity, and outgrowing of axons [31].

### 3.3. Salvia officinalis

*Salvia officinalis* (Sage) is a perennial herb with well-known carminative, antispasmodic, antiseptic, astringent, and antihydrotic properties [85]. The phytocomplexes of *S. officinalis* contain monoterpenes with a broad range of carbon skeletons, including acyclic, monocyclic, and bicyclic compounds, phenolic compounds, diterpenes, triterpenes [86,87]. An alcoholic extract of *S. officinalis* leaf (100 mg/kg i.p.) exhibited an anti-nociceptive effect on cisplatin-induced hyperalgesia in mice. In the formalin test, the aqueous extract effectively suppressed the second phase of pain. The extract even showed stronger benefits than morphine [34]. Vujosevic et al. showed anti-mutagenic effects of *S. officinalis* in a mammalian system in vivo [88], and Keshavarz et al. showed its anti-angiogenic properties for anti-tumor effect in chicken eggs [89].

### 3.4. Walnut

Walnut is one of the traditional anti-tumor, anti-inflammatory, blood purifying, and antioxidant agents. Shabani et al. investigated whether walnut has a neuroprotective property on neurotoxicity induced by cisplatin. Dietary walnut (6%) altered cerebellum- and hippocampus- related behaviors caused by continuous cisplatin injection in male rats. Dietary walnut also ameliorated motor and memory capacities in cisplatin-treated rats. Cisplatin increased, but walnut decreased the latency to nociceptive stimuli [35]. Dietary walnut suppressed mammary gland tumorigenesis in the C(3)1 TAg mouse [90] and growth of implanted MDA-MB 231 human breast cancer cells in nude mice [91]. It has also been demonstrated that walnut reduces growth of prostate cancer [92,93] and colorectal cancer [94].

## 4. Phytochemicals and Medicinal Herbs against Oxaliplatin-Induced Peripheral Neuropathy

Oxaliplatin is a platinum-based anti-neoplastic agent used for treating advanced colorectal cancer [95]. Its cytotoxicity is considered to result from the inhibition of DNA synthesis, similar to that of other platinum complexes. Oxaliplatin forms both intra- and inter-strand crosslinks in DNA, which prevent DNA transcription and replication, resulting in programmed cell death [96]. This chemotherapeutic drug is typically used alongside a combination of folinic acid and 5-fluorouracil (FOLFOX). Oxaliplatin has less ototoxicity and nephrotoxicity than cisplatin; however, oxaliplatin treatment can still cause neurotoxicity and ototoxicity [97].

### 4.1. Curcumin

Al Moundhri et al. showed that oral administration of curcumin (10 mg/kg) reduced drug consistency in the sciatic nerve and prominently ameliorated sciatic nerve injury in oxaliplatin-induced neurotoxicity in rats [24]. Wassem and Parvez showed that curcumin can ameliorate changes in both enzymatic and nonenzymatic antioxidants of mitochondria in vitro. The results reveal the potential of curcumin as a substance that can diminish oxaliplatin-induced peripheral neurotoxicity [98].

### 4.2. Rutin and Quercetin

Rutin (2-(3,4-dihydroxyphenyl)-5,7-dihydroxy-3-{[(2*S*,3*R*,4*S*,5*S*,6*R*)-3,4,5-trihydroxy-6-({[(2*R*,3*R*,4*R*,5*R*,6*S*)-3,4,5-trihydroxy-6-methyloxan-2-yl]oxy}methyl)oxan-2-yl]oxy}-4*H*-chromen-4-one) and quercetin (2-(3,4-dihydroxyphenyl)-3,5,7-trihydroxy-4*H*-chromen-4-one) are polyphenolic flavonoids found in many medicinal herbs and vegetables. They have been reported to have powerful antioxidant, anti-inflammatory, and anti-nociceptive activities. Rutin is water-soluble and is converted to quercetin once it enters the bloodstream [99]. In alcohol-induced neuropathy, quercetin compound showed remarkable anti-nociceptive and neuroprotective effects [100]. In oxaliplatin-treated rats, rutin and quercetin (25, 50, and 100 mg/kg, i.p.) suppressed neuronal contraction and averted development of edema. Moreover, c-Fos, a marker of neuroplasticity, was decreased by rutin- or quercetin- pretreatment [25]. The neuroprotective mechanism of these phytochemicals is connected to its amelioration of mitochondrial dysfunction [98]. Interestingly, quercetin, but not rutin, inhibited azoxymethane-induced colorectal carcinogenesis in rats [101]. Quercetin has been used in clinical trials in cancer patients. The results demonstrated that quercetin can be safely administered by i.v. and that its anticancer properties are detectable [102].

### 4.3. Camellia sinensis (Green Tea)

*Camellia sinensis* is a small tree in which the leaves are used to produce green tea, a popular beverage with therapeutic applications. The key bioactive substances of *C. sinensis* are catechins. A range of studies have demonstrated that these substances have strong anti-inflammatory, antioxidant, and anticancer properties [103]. The therapeutic property of *C. sinensis* was tested against oxaliplatin-induced peripheral neuropathy. Oral administration of green tea extract (300 mg/kg for 6 weeks) forcefully attenuated thermal hyperalgesia and mechanical allodynia; however, it did not avert morphometric or electrophysiological changes [32]. The experimental evidence exhibiting the cancer-preventive activity of green tea is increasing rapidly [104]. Nakachi et al. suggested that consumption of green tea prior to cancer development was markedly associated with improved prognosis of stage I/II breast cancer [105].

### 4.4. Gyeryongtongrac-Bang (Guilongtongluofang in Chinese)

Gyeryongtongrac-Bang is a traditional medicine used to relieve numbness and cold sensation in patients. One hundred twenty colorectal cancer patients who were treated with oxaliplatin were randomly assigned to the Gyeryongtongrac-Bang-treated group (which received aqueous extract from *Ramulus cinnamomi*, *Earthworm*, *Radix astragali*, *Safflower*, *Radix* *angelicae sinensis*, *Ligusticum*, *Spatholobus*, *Radix paeoniae alba*, *Rhizoma curcumae*, and *Licorice* once a day) and the control group (which received placebo) in a double-blind trial. A total of 51.7% patients in the Gyeryongtongrac-Bang-treated group showed neurotoxicity, whereas, it was seen in 70.0% of the placebo-treated group after 4 cycles of treatment. The results suggest that Gyeryongtongrac-Bang can be a potent agent that prevents neurotoxic pain without diminishing oxaliplatin-attributed benefits. Additionally, the development of sensory neurotoxicity was delayed in the Gyeryongtongrac-Bang-treated group [37].

### 4.5. Gyejigachulbu-Tang (Keishikajutsubuto in Japanese)

Gyejigachulbu-Tang is an herbal formula including *Aconiti tuber*, *Atractylodis lanceae rhizome*, *Cinnamomi cortex*, *Glycyrrhizae radix*, *Paeoniae radix*, *Zingiberis rhizoma*, and *Zizyphi fructus*. In our experiment, oral administration of Gyejigachulbu-Tang (200, 400, and 600 mg/kg for 5 days) markedly ameliorated mechanical- and cold-allodynia induced by oxaliplatin treatment. The formula possibly functions through the suppression of spinal glial activation [36]. We confirmed that the extract of *Aconiti tuber* could attenuate both cold and mechanical allodynia similar to Gyejigachulbu-Tang treatment. Interestingly, *Cinnamomi cortex* and coumarin, a phytochemical from *C. cortex*, also attenuated cold allodynia induced by oxaliplatin treatment in rats (unpublished data). These results suggest that both *A. tuber* and *C. cortex* have neuroprotective properties against oxaliplatin-induced neuropathy, thereby playing a major role in the anti-allodynic effect of Gyejigachulbu-Tang.

### 4.6. Jesengsingi-Hwan (Goshajinkigan in Japanese)

Jesengsingi-Hwan is a traditional herbal formula widely used in Asia. It contains 10 different herbs comprising *Achyranthis bidentatae radix*, *Alismatis rhizome*, *A. tuber*, *C. cortex*, *Corni fructus*, *Dioscorea opposita rhizoma*, *Plantaginis semen*, *Poria alba*, *Moutan cortex*, and *Rehmannia viride radix*. Recently, the beneficial properties of Jesengsingi-Hwan on CIPN have been widely prospected. In a murine study, Ushino et al. showed that Jesengsingi-Hwan can reduce CIPN without influence on anti-cancer potency [41]. Kono et al. examined a preventive effect of Jesengsingi-Hwan on chronic oxaliplatin-induced hypoesthesia in rats. Oral administration of Jesengsingi-Hwan (0.3 or 1.0 g/kg, 5 times a week for 8 weeks) ameliorated abnormal sensations and histological damage to the sciatic nerve [42]. In a retrospective clinical study, Kono et al. examined the benefits of Jesengsingi-Hwan on oxaliplatin treatment involved in peripheral neuropathy. In the study, the administration of Jesengsingi-Hwan (7.5 g/day) reduced the neurotoxicity of oxaliplatin in colorectal cancer patients [44]. Later, they again reported that daily oral administration of Jesengsingi-Hwan (7.5 g/day) has the capability to delay the development of grade 2 or greater oxaliplatin-induced peripheral neurotoxicity without impairing FOLFOX efficacy in a randomized phase II study [45]. Yoshida et al. also assessed the effects of Jesengsingi-Hwan for oxaliplatin-induced peripheral neurotoxicity in colorectal cancer patients. Twenty-nine colorectal cancer patients received ≥4 weeks of Jesengsingi-Hwan (2.5 g orally 3 times daily before or between meals for a total of 7.5  g/day) for oxaliplatin-induced peripheral neuropathy during chemotherapy. They were compared to 44 patients who had not received Jesengsingi-Hwan during the same period. A Kaplan-Meier analysis showed that Jesengsingi-Hwan could prevent exacerbation of oxaliplatin-induced peripheral neuropathy [46]. Hosokawa et al. assessed the preventive properties of Jesengsingi-Hwan on oxaliplatin-induced neurotoxicity in colorectal cancer patients and found that in the Jesengsingi-Hwan-treated group, 50% of oxaliplatin-induced peripheral neuropathy was prevented without diminishing chemotherapy efficacy [47].

### 4.7. Hwanggiomul-Tang (Ogikeishigomotsuto in Japanese)

In Japan, a single case study was reported using Hwanggiomul-Tang for oxaliplatin-induced neuropathic pain. Hwanggiomul-Tang is an herbal mixture containing *Zingiberis hizome*, *Jujubae fructus*, *Paeonia alba radix*, *C. cortex*, and *Astragalus membranaceus radix*. The case study of a 59-year old man with recurrent colon cancer suggests that this herbal formula may be useful to reduce or prevent chronic cumulative neurotoxicity due to oxaliplatin [38].

## 5. Phytochemicals and Medicinal Herbs against Paclitaxel-Induced Peripheral Neuropathy

Paclitaxel is a member of the taxane family of drugs used to treat ovarian, breast, lung, esophageal, prostate, bladder, and pancreatic cancer as well as Kaposi's sarcoma and melanoma [106]. This tubulin-targeting drug protects the microtubule polymer from disassembly by stabilizing it. The stabilization triggers apoptosis through inhibition of mitosis [107]. Paclitaxel-treated cells thus have defects in cell division, chromosome segregation, and mitotic spindle assembly. Paclitaxel can induce phenomena of sensory peripheral neuropathy, such as paresthesia and numbness in the extremities [108]. Symmetrical loss of sensation is also a frequent occurrence. These neuropathy symptoms limit the use of paclitaxel.

### 5.1. Cannabidiol

Cannabidiol (2-[(1*R*,6*R*)-6-isopropenyl-3-methylcyclohex-2-en-1-yl]-5-pentylbenzene-1,3-diol) is a key phytochemical accounting for approximately 40% of the *C. sativa* extract [109]. This phytocannabinoid is known to have various medical applications on the basis of clinical reports showing the lack of side effects and particularly a lack of psychoactivity with anti-nausea, anti-psychotic, anti-anxiety, and anti-convulsive properties [110]. Cannabidiol (2.5–10 mg/kg, i.p. for 6 days) inhibited paclitaxel-induced neuropathic pain through 5-HT_1A_ receptor signaling without diminishing chemotherapy efficacy or nervous system function in mice [23].

### 5.2. Verticinone

Verticinone ((3β,5α)-3,20-dihydroxycevan-6-one) is a kind of isosteroidal alkaloid derived from *Fritillaria bulbus*. Xu et al. [26] examined its analgesic effects using neuropathic pain and inflammation models in rats. The experiments showed that hydro-alcoholic extracted verticinone (1.5–3 mg/kg, p.o.) has a relatively constant analgesic effect in paclitaxel-induced neuropathy; further, the analgesic effect of morphine was decreased after repeated medication. Authors believe verticinone is an anodyne with low tolerance.

### 5.3. Jesengshingi-Hwan

Bahar et al. reported that paclitaxel-induced allodynia was markedly prevented by Jesengshingi-Hwan (1 g/kg, p.o. daily) in mice, although Jesengshingi-Hwan could not suppress cancer-induced allodynia [43]. Andoh et al. [111] also reported that Jesengsingi-Hwan (0.1–1.0 g/kg, p.o.) markedly inhibited paclitaxel-induced mechanical allodynia in mice. Authors predicted that *Achyranthis radix* and *P. semen* in Jesengsingi-Hwan may block the aggravation of paclitaxel-induced neuropathic pain. Yamamoto et al. reported that Jesengsingi-Hwan treatment was beneficial for the treatment of paclitaxel-induced neuropathic pain in eighty-two patients enrolled in clinical trials. The investigators believe its preventive effect may be more potent if it is administered from the start of chemotherapy for breast, colorectal, or gynecological cancer patients [48].

### 5.4. Jakyakgamcho-Tang (Shakuyakukanzoto in Japanese)

Jakyakgamcho-Tang is an herbal mixture of *Paeoniae radix* and *Glycyrrhizae radix*. A mouse study found that this combination (1.75 mg/mouse) remarkably attenuated paclitaxel-induced hyperalgesia and allodynia [39]. Through retrospective case analysis on 23 ovarian cancer patients, Fujii et al. [40] concluded that Jakyakgamcho-Tang (7.5 g/day, p.o. for 8 days) has a remedial value in neuropathic pain after paclitaxel and carboplatin combination chemotherapy. Authors suggest that paclitaxel combination chemotherapy with Jakyakgamcho-Tang taken orally is a safer and more tolerable way to reduce pain in epithelial ovarian carcinoma.

## 6. Conclusions and Perspectives

Despite the high incidence of CIPN and its dose-limiting effects, there are no current treatments or preventive options for CIPN with conclusive efficacy and safety data. The lack of effective therapeutic methods for CIPN has boosted the need for the use of medicinal herbs and phytochemicals; these have gained increasing attention as a major form of alternative therapy because they are convenient, economical, effective, safe, and therapeutic. Most recently, a number of phytochemicals and herbal medicines have shown potential for protective benefits for CIPN. Owing to the diverse mechanisms of CIPN, the results of phytotherapy using phytochemicals or herbs contributing to the multiple targets of CIPN seem to be encouraging.

To date, however, many of the therapeutic mechanisms of these phytotherapies remain unclear. For example, significant roles of transient receptor potential (TRP) channels for CIPN development have been discovered, but the link between phytotherapy and TRP channels is mostly unknown. An increasing number of studies report the involvement of TRP channels including TRPA1, TRPM8, TRPV1, and TRPV4 in CIPN [112,113,114,115]. Recent studies found that Jesengsingi-Hwan prevents oxaliplatin-induced peripheral neuropathy through the functional alteration in TRPA1 and TRPM8 [116] and can reduce paclitaxel-induced peripheral neuropathy by suppressing TRPV4 expression [117]. Moreover, the TRPV1-mediated anti-cancer effects of cannabidiol have been reported in multiple cancer cell lines, including breast [118], lung [119,120], and colon [121]. Inflammatory and neuro-immune responses also play important roles in the development and progression of CIPN [122]. Phytotherapies, especially cannabinoids, have significant anti-inflammatory and immunomodulatory properties [123]. More studies are necessary to further our understanding of the involved mechanisms such as TRP channels and immunomodulation. In addition, the dose of several phytochemicals and medicinal herbs for the treatment of animals appears high. At high doses, some of them can induce toxicity in the liver or kidneys. Thus, the dose should be interpreted and verified for toxicity. It is essential to find the maximum dose of phytochemicals and herbs for use in humans.

In conclusion, because of their multitarget, multilevel, and integrated benefits, medicinal herbs seem to be a feasible method for the management of CIPN. Phytochemicals, medicinal herbs, and their formulas could be considered for the treatment of CIPN. However, their curative usability should be examined in well-designed clinical trials. In addition, their reciprocal effects with other drugs in humans should be examined in detail.

## Figures and Tables

**Table 1:** Phytochemicals against CIPN.

Phytochemical	Dose	Chemotherapy	Effects	Refs.
Auraptenol 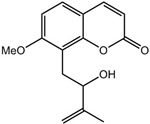	0.05–0.8 mg/kg	Vincristine in mice	Suppresses mechanical hyperalgesia and alteration of behavioral and biochemical changes	[22]
Cannabidiol 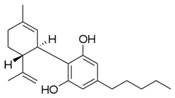	2.5–10 mg/kg	Paclitaxel in mice	Inhibits neuropathic pain through 5-HT_1A_ receptor signaling without diminishing chemothferapy efficacy or nervous system function	[23]
Curcumin 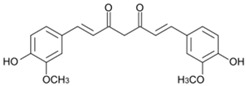	10 mg/kg	Cisplatin or oxaliplatin in rats	Reverses the alterations of neurotensin levels in the plasma, protects the sciatic nerve from injury, and reduces drug absorption in the sciatic nerve	[24]
Rutin 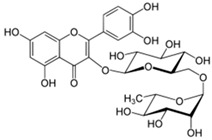 & quercetin 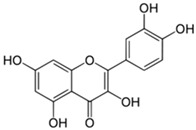	25–100 mg/kg	Oxaliplatin in rats	prevent the shrinkage of neurons and inhibit edema	[25]
Verticinone 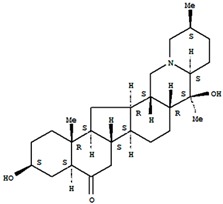	1.5–3 mg/kg	Paclitaxel in rats	Has a relatively constant analgesic effect; the analgesic effect of morphine was decreased after repeated medication	[26]
Xylopic acid 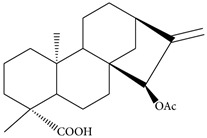	10–100 mg/kg	Vincristine in rats	Has anti-allodynic and anti-hyperalgesic properties	[27]

**Table 2:** Medicinal herbs against CIPN.

Herbs	Dose	Chemotherapy	Effects	Refs.
*Acorus calamus*	100–200 mg/kg	Vincristine in rats	Attenuates symptoms of neuropathy through serotonin 5-HT_1A_ receptors	[28]
*Butea monosperma*	200–400 mg/kg	Vincristine in rats	Reverses alterations of behavioral, biochemical, and histopathological changes	[29]
*Ginkgo biloba* L.	50–150 mg/kg	Vincristine in rats	Decreases paw-withdrawal frequency to cold stimuli and increases the threshold to mechanical stimuli Suppresses NF-κB activation and production of TNF-α and NO Inhibits axonal degradation Improves axonal transportation	[30]
	100–200 mg/kg	Cisplatin in rats, mice, guinea pigs	Protects the inner ear from ototoxicity	[31]
*Camellia sinensis*	300 mg/kg	Oxaliplatin in rats	Alleviates mechanical allodynia and thermal hyperalgesia, but does not prevent morphometric or electrophysiological alterations	[32]
*Ocimum sanctum*	100–200 mg/kg	Vincristine in rats	Attenuates neurotoxicity with the decline in calcium levels and oxidative stress	[33]
*Salvia officinalis*	100 mg/kg	Cisplatin in mice,	Suppresses a second phase of cisplatin-enhanced pain in the formalin test	[34]
*Walnut*	6% of diet	Cisplatin in rats	Inhibits an alteration in performance of hippocampus- and cerebellum-related behaviors	[35]
*Xylopia aethiopica*	30–300 mg/kg	Vincristine in rats	Has anti-allodynic and anti-hyperalgesic properties	[27]

**Table 3:** Herbal formulas against CIPN.

Herbal Formula	Herbs Composition	Chemotherapy	Effects	Refs.
Gyejigachulbu-Tang	*Aconiti tuber*, *Atractylodis lanceae rhizome*, *Cinnamomi cortex*, *Glycyrrhizae radix*, *Paeoniae radix*, *Zingiberis rhizoma*, *Zizyphi fructus*	Oxaliplatin in rats	Attenuates cold and mechanical allodynia Suppresses spinal glia activation	[36]
Gyeryongtongrac-Bang	*Ramulus cinnamomi*, *Earthworm*, *Radix astragali,* *Safflower*, *Radix angelicae sinensis, Ligusticum*, *Spatholobus*, *Radix paeoniae alba*, *Rhizoma curcumae*, *Licorice*	Oxaliplatin in a randomized, double-blind, placebo-controlled trial	Prevents sensory neurotoxicity and delays its onset	[37]
Hwanggiomul-Tang	*Zingiberis hizome, Jujubae fructus, Paeonia alba radix, Cinnamomi cortex,* *Astragalus membranaceus radix*	A case study of oxaliplatin-treated 59-year-old man with recurrent colon cancer	Prevents chronic cumulative neurotoxicity	[38]
Jakyakgamcho-Tang	*Paeoniae radix, Glycyrrhizae radix*	Paclitaxel in mice	Relieves allodynia and a hyperalgesia	[39]
		A retrospective case analysis investigated 23 patients with ovarian cancer treated with paclitaxel and carboplatin combination chemotherapy	Reduces pain in epithelial ovarian carcinoma	[40]
Jesengsingi-Hwan	*Achyranthis bidentatae radix, Alismatis rhizome, Aconiti tuber, Cinnamomi cortex, Corni fructus, Dioscorea opposita rhizoma, Plantaginis semen, Poria alba, Moutan cortex, Rehmannia viride radix*	Oxaliplatin in rats	Reduces peripheral neuropathy without influence on anti-cancer potency Ameliorates abnormal sensations and histological damage to the sciatic nerve	[41,42]
		Paclitaxel in mice	Inhibits mechanical allodynia	[43]
		Oxaliplatin in a placebo-controlled double-blind randomized study	Delays onset of grade 2 or greater peripheral neurotoxicity without impairing FOLFOX efficacy with an acceptable safety margin	[44,45]
			Prevents exacerbation of peripheral neuropathy	[46,47]
		A clinical trial enrolling 82 patients	Reduces peripheral neuropathy	[48]
